# STLV-1 as a model for studying HTLV-1 infection

**DOI:** 10.1186/s12977-019-0503-0

**Published:** 2019-12-16

**Authors:** Brice Jégado, Fatah Kashanchi, Hélène Dutartre, Renaud Mahieux

**Affiliations:** 1 International Center for Research in Infectiology, Retroviral Oncogenesis Laboratory, INSERM U1111 - Université Claude Bernard Lyon 1, CNRS, UMR5308, Ecole Normale Supérieure de Lyon, Université Lyon, Fondation pour la Recherche Médicale, Labex Ecofect, Lyon, France; 2 Laboratory of Molecular Virology, George Mason University, Manassas, VA USA

**Keywords:** HTLV-1, STLV-1, ATL, Prevalence, Interspecies transmission, Animal model, Therapy

## Abstract

Few years after HTLV-1 identification and isolation in humans, STLV-1, its simian counterpart, was discovered. It then became clear that STLV-1 is present almost in all simian species. Subsequent molecular epidemiology studies demonstrated that, apart from HTLV-1 subtype A, all human subtypes have a simian homolog. As HTLV-1, STLV-1 is the etiological agent of ATL, while no case of TSP/HAM has been described. Given its similarities with HTLV-1, STLV-1 represents a unique tool used for performing clinical studies, vaccine studies as well as basic science.

## Background

The first human oncogenic retrovirus was discovered in the USA, in a T cell line obtained from blood cells of a patient suffering from a disease then called “cutaneous T-cell lymphoma” [1, 2]. Few years earlier, Adult T-cell Leukemia/Lymphoma or ATLL (i.e. an aggressive malignancy of CD4+ T-cells) had been described in Japan [3, 4]. In 1982, Japanese researchers also reported the presence of a retrovirus among ATLL patients. They named it Adult T cell leukemia virus (ATLV). Further work demonstrated that HTLV-1 specific antibodies were present among Japanese ATLL patients, thus allowing identification of the first HTLV-1 endemic area [5]. Later, it was decided to name this virus HTLV-1 for Human T-cell Leukemia Virus type 1.

Few years later, Tropical Spastic Paraparesis/HTLV-1 associated myelopathy (TSP/HAM), a severe neuromyelopathy, was also identified as another disease caused by HTLV-1 [6]. Thus, ATLL and TSP/HAM are the main pathologies present among HTLV-1 infected individuals. It was recently estimated that 5 to 10 million people are infected by HTLV-1 worldwide, although HTLV-1 prevalence is likely to be underestimated. Two to 4% of HTLV-1 carriers will develop either ATLL or TSP/HAM, while most of them will remain asymptomatic [7]. HTLV-1 is endemic in areas such as Japan, central Africa, the Caribbean region and South America [8]. Because HTLV-1 mostly replicates through clonal expansion of infected cells even in asymptomatic carriers [9], its retroviral genome displays a remarkable genetic stability. HTLV-1 molecular epidemiology studies have been carried out throughout the world. The very low genetic variability allowed identification of different HTLV-1 subtypes. All but one of these subtypes, i.e. Cosmopolitan subtype A that is present all over the world, are specific to a given African or Asian region [8]. ATL cases were described in HTLV-1 carriers infected by HTLV-1 subtype A but also subtype B and subtype C [10, 11], thus suggesting that ATL occurrence is not linked to the most frequent HTLV-1 subtype. Of note, HTLV-1 subtype B and subtype C lack p12 and/or p30 auxiliary protein. Whether the lower ATL frequency in type B and C infected individuals is linked to the absence of these proteins remains to be determined.

In 1982, lymphocytes from a Japanese monkey (*Macaca fuscata*) were co-cultured with chronically and productively infected T-cells from the MT-2 cells, an HTLV-1-transformed cell line. This allowed the authors to obtain a simian cell line persistently infected by HTLV-1, thus suggesting that Japanese monkeys might be susceptible to HTLV-1 natural infection [12]. Later, seroepidemiological studies were performed in Japan and demonstrated that many Japanese monkeys were infected by HTLV-1-like viruses [13]. Sera from New World Monkeys (NWM), Old World Monkeys (OWM) and Apes were then tested and revealed the presence of antibodies reacting against HTLV-1 antigens. Such antibodies were detected in OWM and Apes, but not in NWM, suggesting endemicity of HTLV-1-related viruses in African and Asian monkeys, but not in American animals [14]. Sequence analyses characterized these viruses as Simian T-cell Leukemia Viruses (STLVs) [15, 16]. To date, it is well established that Old World Non-Human Primates (NHPs) and Apes are naturally infected with a great variety of STLV-1 viruses and that HTLV-1 appeared in Humans following STLV-1 cross-species transmission approximately 27,300 years ago (95% CI 19,100–35,500) in Africa, even if interspecies transmission episodes still occur [17–19]. Given the high degree of similarity between HTLV-1 and STLV-1 sequences, it was suggested to cluster these viruses in the single PTLV (Primate T lymphotropic virus) family [20–22]. Because STLV-1 induces ATLL in naturally infected NHPs [23, 24], and even if some auxiliary proteins are lacking [25], it represents a suitable tool that contributes to our understanding of HTLV-1 pathogenesis. This review will compare HTLV-1 and STLV-1 retroviruses from different aspects and will focus on the use of STLV-1 as a model of HTLV-1 infection.

## STLV-1 epidemiology

Around 132 non-human primate species represent Old World Monkeys (OWM). They are divided in two subfamilies, *Cercopithecinae* and *Colobinae,* distributed in African and Asian continents [26].

**Fig. 1:**
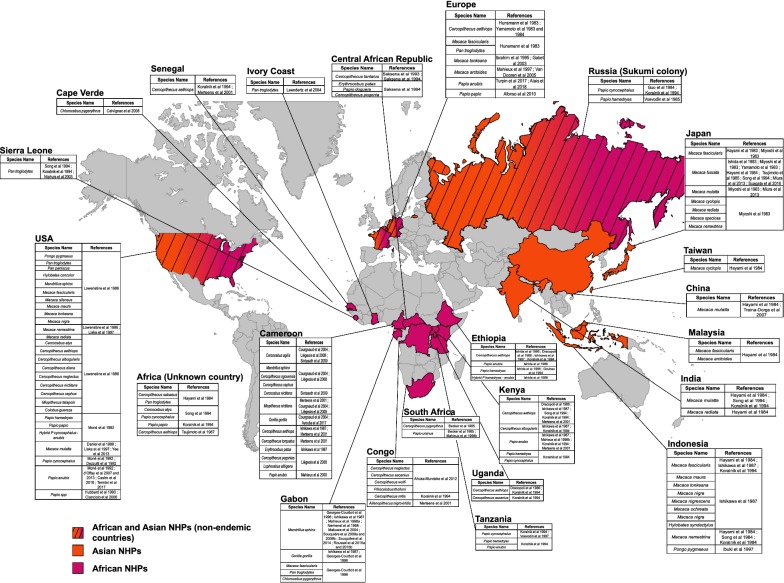
Epidemiology of Simian T-Leukemia Virus Type-1 in wild-caught or captive non-human primates (NHPs) from Asia and Africa. All studies which reported STLV-1 infection in NHPs are listed. Orange and purple colors represent Asian and African STLV-1 infected NHPs, respectively. Countries with both colors and hatching represent Asian and African NHPs hosted in geographical areas where they are not naturally present

To determine which simian species carry STLV-1, seroepidemiological studies were performed using kits that had been previously developed for the detection of anti-HTLV-1 human antibodies, as well as by PCR (Fig. 1). Sera from Japanese monkeys were tested, and 25% scored seropositive. As in humans, STLV-1 incidence increased with age and was higher in females than males. Other species were tested later. A high seroprevalence was observed in African Green monkeys (AGM). Two studies then reported STLV-1 infection in captive Old World NHPs and Apes [27, 28]. Ishikawa et al. [29] performed an STLV-1 survey using 567 NHPs’ blood samples covering 30 species caught in the wild or kept in zoos, institutes or private owners from Kenya, Gabon, Ghana, Cameroon, Ethiopia and Indonesia. STLV-1 was detected in African Green monkeys and Sykes’ monkeys, in Olive baboons, Patas monkeys, Mandrills and Gorillas. STLV-1 was also found in different species of macaques from Indonesia, with a seroprevalence ranging from 11 to 25%. Other studies reported natural STLV-1 infections in AGM, Vervet monkeys and among baboon species (*Papio anubis*, *Papio hamadryas*, *Papio papio* and *Papio cynocephalus*) originating from South Africa and Ethiopia [30–33]. As in Japan, the infection status positively correlates with age, and disease incidence is higher in females than males. Other seroepidemiological studies were also performed [34–44] (Fig. 1). Thirty-one Old World NHP species were reported as naturally infected with STLV-1 [33, 45–50].

STLV-1 sequence analyses were then performed in order to determine relationship between STLV-1 and HTLV-1 and whether HTLV-1 originated from a non-human primate virus.

## STLV-1 phylogeny

Since the first publication of a complete HTLV-1 proviral genome [51], phylogenetic studies enabled to identify several HTLV-1 subtypes: Cosmopolitan subtype A, which is found all over the world; subtypes B, D, E, F, G, which are restricted to Central Africa; and Australo-Melanesian subtype C which is the most divergent HTLV-1 subtype [8]. Based on molecular clock and phylogenetic analyses, origin of HTLV-1 subtypes A, B, D, E was inferred in a time frame of 27,300 ± 8200 years, whereas subtype F arose more than 10,000 years ago.

In 1984, Watanabe et al. [52] demonstrated similarities between restriction maps obtained using HTLV-1 from Robert Gallo’s laboratory or using Japanese simian Adult T-cell Leukemia Virus (ATLV). These results suggested that HTLV-1 and simian ATLV shared a common ancestor. Other studies reported that HTLV-1 and STLV-1 from Japanese monkeys, Red-faced monkeys, Pig-tailed monkeys, AGM, Chimpanzees and baboons (*Papio cynocephalus*) had the same genomic organization i.e. *LTR*-*gag*-*pol*-*env*-*pX*-*LTR* [15, 20]. Sequence analyses comparing Pig-tailed (Asian NHP) and AGM (African NHP) STLV-1 sequences to HTLV-1 revealed 90% and 95% identity respectively. These results suggested that (1) STLV-1 could be separated into two subgroups: Asian and African and that (2) HTLV-1 originated from the African STLV-1 subgroup [16].

Phylogenetic studies revealed that HTLV-1 subtype B is very closely related to STLV-1 strains infecting chimpanzees (98% identity), Allen’s swamp monkeys (around 96% identity) and gorillas from Zaïre, Central African Republic and Cameroon [45, 53–55]. STLV-1 strains infecting *Mandrillus sphinx*, *Cercopithecus cephus, C. agilis, C. pogonias, G. agilis* and *C. nictitans* share close relationships with HTLV-1D and -F from Cameroon and Gabon [49, 56–58]. Regarding HTLV-1 subtype E, the *Env* region clusters with STLV-1 isolated from two baboon species, *Papio ursinus* and *Papio cynocephalus* [59]. No data has been so far reported about a simian counterpart of HTLV-1G and HTLV-1A. Altogether, the diversity of STLV-1 strains found in different NHPs species and related to a given HTLV-1 subtype from the same geographical areas is strongly supporting the concept of multiple cross-species transmissions between NHPs but also from NHPs to humans.

Most divergent STLV-1 strains were described in Asian *Macaca tonkeana* (living in Indonesia) and *Macaca arctoides* (living in India, Thailand and China) [60–62]. *Macaca tonkeana* virus is related to the most divergent HTLV-1 subtype C that is present in Melanesia and Australia. Molecular clock data inferred STLV-1 introduction around 156,000 to 269,000 years ago on the Asian continent [59]. These results suggest that macaque infection with STLV-1 might have led to the emergence of HTLV-1 in Asian human population.

Finally, Calvignac et al. [63] demonstrated that STLV-1 sequences could be amplified from bones samples originating from an early 20th century *Chlorocebus pygerythrus* sample. Therefore, it should now be possible to use this technique to determine STLV-1 virus evolution over time using available Egyptian or Asian NHP mummies.

## STLV-1 interspecies transmission

Prevalence of HTLV-1 may reach 1 to 40% in adults depending on age, sex and geographic location [8]. It is well known that HTLV-1 can be transmitted under different routes: sexual, mother-to-child and contact with infected blood. However, STLV-1 transmission occurs mostly through aggressive contacts instead of mother to infant or sexual transmissions [64–68], even if sexual transmission of STLV-1 is more important in NHPs such as vervet [40].

## STLV-1 associated-disease in naturally infected animals

As it is the case for HTLV-1-infected individuals, most STLV-1-infected monkeys remain lifelong asymptomatic hosts [69]. For some unexplained reasons, TSP/HAM cases have never been observed in infected NHPs, even when those animals were living in animal facilities for a long period. Phylogenetic studies performed using samples from an African human TSP/HAM patient showed that the viral sequence was highly related to an STLV-1 sequence obtained from asymptomatic West-African sooty mangabey [70]. Other strains obtained from HTLV-1 African TSP/HAM patients also clustered with STLV-1 strains obtained from asymptomatic animals [71, 72]. It is well established that there is no specific mutation in HTLV-1 genome that would be associated with a given disease. Altogether, these data suggest that the lack of TSP/HAM described cases in NHPs might only be linked to the mode of viral transmission rather than the age of infection.

On the contrary, a number of ATLL-like diseases sharing clinical and pathological features with human ATLL were reported in NHPs [24, 69, 73–79]. The first report was made in STLV-1 infected macaques which developed malignant lymphoma [80]. Subsequent studies reported similar symptoms in captive *Papio anubis*, Gorillas and AGM [75–78, 81, 82]. In a recent study, Tax-positive cells were detected in lymphoid and non-lymphoid organs, mesenteric and axillary lymph nodes and lung, but not in the blood from an infected *Papio anubis* suffering from ATL [24]. In that case, skin lesion biopsies also showed a massive dermal, hypodermic and muscular cell infiltrates of positive CD3+ CD25+ T cells, as described in human ATL.

## Using STLV-1 infected animals

### After natural STLV-1 infection

Given the high degree of sequence similarities between STLV-1 and HTLV-1 genomes and the fact that both viruses cause ATL, STLV-1 infected NHPs (Japanese macaques, *Mandrillus sphinx* and *Papio anubis*) have been used for performing molecular studies [79, 83–89] (Table 1). As HTLV-1, STLV-1 infection is mostly occurring in CD4+ T-cells, although STLV-1 Tax expression was also detected in bone marrow hematopoietic stem cells in vivo, and viral DNA was retrieved in all myeloid and lymphoid cells derived from these infected progenitors [86].

**Table 1:** STLV-1 naturally or experimentally infected non-human primates (NHPs) described in published biological studies

Studies	Natural STLV-1 infection	STLV-1 inter-NHPs transmission	Experimental HTLV-1 infection
Mechanisms of (co-)infection : retroviral replication	Miura et al. [79]	Dube et al. [94]	Kazanji et al. [96]
	Ma et al. [83]	Voevodin et al. [82]	Kazanji et al. [97]
	Castro et al. [84]	Voevodin et al. [93]	Kazanji et al. [98]
	Termini et al. [85]	Voevodin et al. [32]	Mortreux et al. [99]
	Furuta et al. [86]	Voevodin et al. [53]	Debacq et al. [100]
Drugs and vaccine treatments	Yee et al. [87]	McGinn et al. [95]	Heraud et al. [101]
	Souquière et al. [88]	Pise-Masison et al. [102]	
	Souquière et al. [90]	Valeri et al. [103]	
	Souquière et al. [111]	McGinn et al. [104]	
	Sugata et al. [89]		
Cytotoxic response	Turpin et al. [24]		
	Afonso et al. [92]		

STLV-1 infection mechanisms, experimental treatments and immune response were analyzed in several NHP species

STLV-1 natural infection leads to Tax and SBZ (simian equivalent of HBZ) expression. Simian SBZ and Tax amino-acid sequences are highly similar to human HBZ and Tax (see Tables 2 and 3). These viral proteins also display activating properties on viral LTR and NF-κB signaling pathways. As an example, a high STLV-1 proviral load (PVL) is linked to IL-2, IL-6, IL-10, IFNγ and TNF-α elevated expression in asymptomatic STLV-1-infected *Mandrillus sphinx* [90]. Given well-established results published in the HTLV-1 situation, this is likely due to STLV-1 Tax expression, although this hypothesis has not been formally demonstrated. IL-2 and IFNγ results were also obtained in asymptomatic STLV-1-positive *Macaca mulatta* [87], while anti IFNγ and TNF-α responses against Tax expressing cells were also observed in STLV-1 infected baboons [85]. STLV-1 infection also promotes CTL response against STLV-1 Tax protein [84, 85].

**Table 2:** Amino acid sequence comparison of HTLV-1 HBZ vs. STLV-1 SBZ

	HTLV-1a ATK	HTLV-1b EL
HTLV-1a ATK	–	74.27%
HTLV-1b EL	74.27%	–
STLV-1 *Papio anubis*	83.01%	71.36%
STLV-1 Mf5	75.71%	61.43%

ATK belongs to HTLV-1 A cosmopolitan subtype, EL to HTLV-1 B subtype, STLV-1 *Papio anubis* was obtained from an African NHP, while STLV-1 Mf5 was obtained from an Asian NHP (*Macaca fuscata*)

**Table 3:** Amino acid sequence comparison of HTLV-1 Tax vs. STLV-1 Tax

	HTLV-1a ATK	HTLV-1b EL
HTLV-1a ATK	–	97.26%
HTLV-1b EL	97.26%	–
STLV-1 *Papio anubis*	96.03%	95.74%
STLV-1 Mf5	92.92%	93.31%

ATK belongs to HTLV-1 A cosmopolitan subtype, EL to HTLV-1 B subtype, STLV-1 *Papio anubis* was obtained from an African NHP, while STLV-1 Mf5 was obtained from an Asian NHP (*Macaca fuscata*)

Interestingly, TCF1 and LEF1, two T-cell specific proteins, prevent Tax effect on viral LTR. Their expression is high in thymocytes and thus counteract STLV-1 replication in thymus. On the opposite, their expression and thus their effect is down-regulated in peripheral blood T-cells (both in human and simian cells), thanks to a Tax effect on STAT5a. This might explain why Tax is more potent in these cells, and why HTLV-1 induces ATL in the periphery [83].

Depending upon STLV-1 strain, SBZ protein sequence is highly similar or contain insertions and deletion compared to HBZ (see Table 2). Nevertheless, in both cases, animals can develop ATL [24, 79]. This might be due to conservation of the N-terminal region as well as of C-terminus basic leucin zipper domain between human and simian viral proteins.

As its human counterpart, STLV-1 replication occurs through clonal expansion of infected cells, both in asymptomatic and ATL animals [24, 79]. Antiviral therapy based on the use of azidothymidine (AZT) combined with interferon-α (IFN-α) improves the survival rate of ATL patients suffering from acute and chronic/smoldering forms. A confirmation clinical trial using these compounds was reported in an STLV-1 infected *Papio anubis* suffering from ATL. The animal was treated with a combination of AZT and interferon-α. However, and contrary to human ATL, no clinical improvement was observed. It would now be interesting to determine post-mortem whether, this absence of remission was linked to *p53* mutation already present when treatment started as shown in human ATL cases who were not responding to AZT [91].

Given the fact that treating ATL patients is difficult, and because an elevated PVL is a characteristic of ATL, a study tested whether PVL decreases when valproate and AZT were delivered to asymptomatic STLV-1-infected animals [92]. This was indeed the case and it was associated to an increased anti-Tax CTL response, thus confirming the importance of immune response for controlling viral infection [92]. In another study, STLV-1 infected asymptomatic Japanese monkey were inoculated with mogamulizumab (anti-CCR4), a component that is also used for human relapsed ATL cases. This led to a strong reduction of STLV-1 proviral load [79, 89]. Altogether, these results support the fact that STLV-1 infected animals represent a useful tool for testing drugs.

Finally, a recent study was performed in two asymptomatic STLV-1-infected animals. This showed that immunization using recombinant vaccinia viruses expressing either Tax-22 (which cannot activate the NF-kB pathway) or an HBZ LL/AA mutant (which is partially impaired for blocking Tax ability to induce transcription) was linked to a temporary decrease of STLV-1 PVL [89].

### After STLV-1 interspecies transmission

A limited number of reports described STLV-1 inter-simian species transmission [32, 53, 93, 94] (Table 1). In one report and following an unknown mode of transmission, it was shown that baboons accidentally infected with a rhesus macaque STLV-1 virus, developed leukemia/lymphoma at a high frequency [93]. This is the only reported case suggesting that inter-simian species transmission might impact viral pathogenesis. Experimental infection of pig-tailed macaques with sooty mangabey STLV-1 was also tested. Animals maintained low antibody titers and displayed a high mortality rate without any identified cause [95]. Finally, another work reported tantalus and patas animals artificially infected with STLV-1 from other species. All animals became infected, as shown by PCR results, even if one stayed seronegative due to mutations in the genome [94]. Why were these *pol* mutant viruses still able to infect animals remains unexplained.

### After artificial HTLV-1 infection

Finally, given the high degree of similarity between HTLV-1 and STLV-1 genomes and the abundance of molecular tools available in the HTLV-1 field, some laboratories decided to use the HTLV-1 molecular clone or HTLV-1 infected cells to perform studies in non-human primates (Table 1). Artificial infection after inoculation of HTLV-1 to primates provides an inestimable tool to study primo-infection and viral dissemination, in vivo, a process that is inaccessible in humans. HTLV-1 infection of *Saimiri sciureus*, i.e. non-human primates that are not naturally infected with STLV-1 [96], demonstrated that lymphoid organs represent the major viral reservoir [97]. As in HTLV-1 infected humans and STLV-1 naturally-infected animals, IL-2, IL-10, IFNγ levels also increased after HTLV-1 infection [98]. In *Saimiri sciureus*, the virus also replicates through clonal expansion after having used reverse transcription (RT) at the initial stages [99] and it causes ATL [100]. As in baboons treated with AZT/IFN [24], arsenic combined to IFN-α was not able to lead to HTLV-1 proviral load reduction, even if the number of circulating ATL flower cells decreased for some unexplained reason [101].

Studies were also performed in pig-tailed and rhesus macaques inoculated with autologous cells previously transfected with the HTLV-1 ACH molecular clone [102–104]. Following infection with wild-type HTLV-1, pig-tailed macaques developed a series of extremely aggressive diseases that were different from ATL. These results therefore suggest that this animal model cannot be used for studying events that are resulting from HTLV-1 infection.

Consequences of rhesus macaque infection with the same molecular clone were different since animals remained asymptomatic. HTLV-1 p12 and p8 proteins have been shown previously to increase NFAT activity, IL-2 production and STAT-5 activity, while p30 controls viral expression at the post-transcriptional level in vitro (for a review, see [105, 106]). Thus, this simian model was useful to investigate the role of p12, p13, and p30 auxiliary proteins in vivo [102, 103]. This allowed researchers to show that p12 and p30 are required for allowing HTLV-1 presence and replication in dendritic cells [103], while p12 and p8 are necessary for allowing a viral resistance to CTL responses. These studies provided the first in vivo evidence on the mechanisms that HTLV-1 uses to establish chronic infection and on the crucial role of myeloid cells in that process.

Interestingly, the authors also demonstrated that the results obtained in rhesus macaques were different from those obtained in rabbits infected with the same viral clones, thus reinforcing the fact that NHPs are the more relevant system for studying HTLV-1 pathogenesis.

## PTLV retroviral coinfection in NHPs and in humans

In addition to STLV-1, other retroviruses, i.e. Simian Immunodeficiency Virus (SIV) and Simian Foamy Virus (SFV) infect NHPs. Cases of natural coinfection have been reported both in humans and in NHPs: HTLV-1/HIV-1, HTLV-1/HFV, STLV-1/SFV or STLV-1/SIV-1 [67, 107–115]. HIV-1/HTLV-1 coinfection leads to significant increase of HTLV-1 PVL as well as on a possible delay in HIV-1 pathogenesis in humans [107, 108, 116]. Anti-HIV-1 therapy promotes an increase in HTLV-1 PVL in HIV-1/HTLV-1 coinfected carriers. These results strongly suggest that both retroviruses compete for CD4+ T-cell infection. However, it is worth noting that opposite results were obtained in other studies [117–121].

Natural STLV-1/SIV-1 co-infection induces the development of a neoplastic disease in sooty mangabey [122] and of a lymphoproliferative disease in AGM [123]. Souquière et al. described pathological manifestations, i.e. infective dermatitis and scabies, in two STLV-1/SIV-1 co-infected mandrills [111], while no clinical sign has been reported previously in STLV-1 naturally infected mandrills [90]. Thus, these symptoms could be due to co-infection. Ongoing experiments should allow us to determine whether STLV-1 clonal expansion impacts SIV replication in vivo.

Finally, blood SFV proviral load from STLV-1/SFV naturally co-infected *Papio anubis,* was recently shown to be much higher compared to SFV mono-infected animals [124]. These results either suggest that cells might be co-infected with both retroviruses, with STLV-1 promoting clonal expansion, or that soluble STLV-1 Tax transactivator enters SFV-infected cells where it promotes viral replication. Ongoing experiments should allow us to answer this question.

Altogether, these data demonstrate that STLV-1 is a useful tool to understand mechanisms of HTLV-1 transmission and ATL pathogenesis. PTLV-1 mono-infected as well as SIV co-infected animals could also be used to develop possible new anti-HTLV-1 clinical approaches and to modify anti-HIV treatment.

## Data Availability

Not applicable.
